# The role of the histoblood ABO group in cancer

**DOI:** 10.4155/fsoa-2015-0012

**Published:** 2016-03-15

**Authors:** Seth K Rummel, Rachel E Ellsworth

**Affiliations:** 1 Clinical Breast Care Project, Windber Research Institute, 620 Seventh Street, Windber, PA 15963, USA; 2 Clinical Breast Care Project, Murtha Cancer Center, 620 Seventh Street, Windber, PA 15963, USA

**Keywords:** cancer, histoblood ABO group, rs505922

## Abstract

Since the first link between blood type and cancer was described in 1953, numerous studies have sought to determine whether the histoblood ABO group is associated with tumorigenesis. In 2009, the first significant association between a SNP located within the ABO glycosyltransferase gene and increased risk of pancreatic cancer was reported. Here, we describe the history and possible functions of the histoblood ABO group and then provide evidence for a role of blood group antigens in the most common cancer types worldwide using both blood type and SNP data. We also explore whether confusion regarding the role of blood type in cancer risk may be attributable to heterogeneity within tumor types.

## Abstract (lay abstract)

**Lay abstract:** *ABO* encodes the protein responsible for defining blood groups as A, B, AB or O. Despite over a century of investigation, it is not well known whether the blood group antigens have a function or if they contribute to human health. Over the last 60 years, associations between blood type and cancer risk have been reported, although the data have often been conflicting. To better understand the possible role of the ABO blood group in tumorigenesis, we review the data for the most common tumor types worldwide.

**Figure 1.**
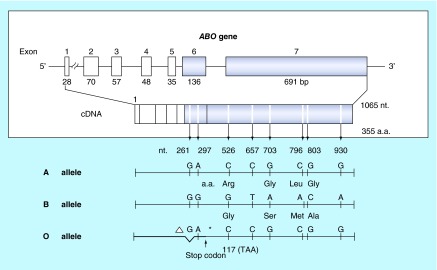
**Structure of the *ABO* gene locus and nucleotide sequences of A, B and O alleles.** Diagram of exon organization of the protein coding sequences (under shaded). Reprinted with permission from the Tokushima Medical Association [11]. ^†^Entirely different deduced a.a sequence in O alleles due to frame-shifting caused by single base pair deletion. a.a.: Amino acid; bp: Base pair; nt.: Nucleotide position.

The ABO blood group system was first described in 1900 by K Landsteiner based on agglutination patterns of red blood cells when blood types from different donors were mixed [1]. The ABO blood group system is based on expression of two antigens, A and/or B on the surface of the red blood cell; because expression of these antigens is codominant, patients may have type A, type B or type AB expression patterns. Lack of expression of either antigen results in the O phenotype. Blood group frequencies vary globally with type O, whose frequency approaches 100% among the indigenous populations of Central and South America, being most common, followed by type A, more common in central and eastern Europe, B, more common in China and India and AB which is more frequent in Japan, China and Korea [2].

The ABO blood group has been associated with a number of diseases or hemostatic complications. For example, meta-analyses have detected associations between increased risk of coronary heart disease, and venous thromboembolism and the nontype O blood types [3,4], while the O blood group is protective against falciparum infection and is associated with reduced risk of severe malaria [5]. In contrast, the O blood group has been implicated with increased risk of hemorrhage [6]. The AB phenotype has been associated with increased risk of preeclampsia [7].

Associations have also been made between the ABO blood group and cancer. The first report describing a link between the A antigen and increased risk of stomach cancer was published in 1953 [8]. Over the next six decades, numerous studies attempting to validate these results or identify associations with other tumor types have been performed with mixed results. In 2009, a link between the ABO blood group and risk of pancreatic cancer was established, not by evaluating blood groups, but through a genome-wide association study (GWAS) linking an SNP in the first intron of *ABO* with increased risk [9]. How this SNP contributes to increased risk of pancreatic cancer and whether this SNP or any other variants of the ABO blood group system are associated with other types of cancers remains unclear. Here, we present a comprehensive review of the literature evaluating the link between the ABO blood group and cancer.

## The histoblood group ABO

The antigens of the ABO blood group are oligosaccharides: the A phenotype is defined by the sugar N-acetylgalactosamine (GalNAc) and B by galactose (Gal). These sugars are transferred to a precursor antigen known as the H antigen controlled by a single gene, *ABO*. Located on chromosome 9q34.2, *ABO* is comprised of seven exons with DNA variants altering the gene's enzymatic activity (Figure 1). Four nonsynonymous variants at nucleotides 526, 703, 796 and 803 are responsible for the differences resulting in either α1-3 N-acetylgalactosaminyltransferase (A allele) or α1-3 galactosyltransferase activity (B allele). A single base pair deletion at base pair 261 leads to a truncated, nonfunctional protein without enzymatic activity (O allele) [10].

Other than determining blood phenotypes, the function of *ABO* is not clear. To date, no lethal mutations in *ABO* have been identified and failure to express a functional protein is compatible with life, suggesting that the blood group antigens may not have a function [12], yet polymorphisms controlling the A and B phenotypes are conserved across multiple primate species suggesting that these antigens may affect the fitness of humans and other primates [13]. In addition, ABO antigens are expressed on cell types other than red blood cells, including platelets, vascular endothelial cells, mucus secretions and epithelial tissues, where different blood types have been associated with increased risk of disease. For example, antigens are expressed in the mucin of the digestive tract where they may differentially bind to gut pathogens [12]. Expression of blood group antigens on von Willebrand factor alters platelet aggregation; type O individuals, who have ˜25% lower levels of von Willebrand factor, are at risk for increased bleeding but decreased thrombosis [14]. *ABO* may also be associated with cancer risk as the A antigen can be detected in tumor cells from non-A individuals, while glycosylation can lead to conformational changes in proteins such as the epidermal growth factor receptor or alter immune recognition of natural killer cells, conditions that favor tumorigenesis [12].

## ABO blood group & cancer

### Gastric cancer

The first link between the ABO blood group and cancer was described in 1953 [8]. Blood types were evaluated in 3632 patients with stomach cancer from England and Scotland; when compared with controls, there was a significantly higher frequency of the A blood group and lower frequency of O in patients with stomach cancer, suggesting that the A blood group increased risk, while the O blood type was protective. A follow-up paper reported that the B blood group was also found at decreased frequency in patients with stomach cancer [15]; of note, no association between any blood groups and carcinomas of the colon, rectum, bronchus or breast was detected in this English patient population. By 1959, 12 studies had reported an association of the A blood group with increased risk of stomach cancer, with a 21% increased mean incidence of stomach cancer in patients with the A blood type [16], and by 1966, a combined analysis of 71 publications supported an association between blood type A and increased risk for stomach cancer [17]. In a recent meta-analysis (Table 1), the A blood group was associated with an increased risk of gastric cancer compared with non-A blood types (odds ratio [OR] = 1.11; 95% CI: 1.07–1.15) and the O blood group with decreased risk compared with non-O blood types (OR: 0.91; 95% CI: 0.89–0.94); of note, patients with the A blood type were also at higher risk of infection by *Heliobacter pylori*, a risk factor for noncardia gastric cancer [18].

Stomach cancer accounts for 7% of cancers worldwide [24]. While the evidence for an increased risk of developing stomach cancer in patients with the A blood type is convincing, links between blood group and other types of cancers is less clear. A summary of results for the most common cancers worldwide are described below.

### Lung cancer

Lung cancer is the most common cancer worldwide, accounting for 13% of all newly diagnosed cancers [24]. In agreement with the results from Aird *et al*. [8], in a series of 1257 men with lung cancer treated in Wales, no association between blood group and lung cancer was detected [25]. In this study, however, when tumors were subclassified, there was an increased frequency of type A blood type and decreased frequency of type O blood in patients with proximal tumors and *vice versa* with distal tumors. In a recent study, Turkish patients with non-O blood types were at increased risk of developing lung cancer [26]. In contrast, a separate study of Turkish patients found no association between the ABO blood group and either non-small-cell lung carcinoma or small cell lung carcinoma [27]. Decreased survival of patients with lung cancer has been associated with presence of the B allele in patients with either B or AB blood types [28].

ABO antigens are also expressed in mucus of the lung. Studies demonstrate that in non-O blood group patients, there is a loss of the A and/or B antigens from tumor cells [29]. Greater loss of antigens within the tumor cells has been associated with less favorable prognosis [30–33] and in non-small-cell lung carcinoma, loss of the A antigen was associated with shorter time to progression and decreased survival [34–37]. In contrast, other groups have failed to detect an association between loss of blood antigens in tumor tissue and survival [38–40].

### Breast cancer

Breast cancer accounts for 12% of newly diagnosed tumors worldwide [24]. A number of studies have investigated the role of the ABO blood group in breast cancer risk and pathology. Two early publications found no association between ABO blood group and risk of breast cancer in either English or American patient populations [15,41]. More recent studies include the Nurses’ Health Study in USA which found no association between blood group and breast cancer risk or survival [42] and a study from Turkey that found no association between breast cancer risk and any ABO blood types [43]. Two other studies, however, reported that the A antigen was associated with increased risk of developing invasive ductal carcinoma in 166 Greek women, while in a cohort of Icelandic patients, risk of familial breast cancer was associated with the B blood group [44,45]. Two meta-analyses suggest that the A blood group is associated with increased risk of breast cancer [20,23].

As with lung carcinomas, loss of antigen expression has been detected in primary breast tumors and their metastases [46,47]. Loss of antigen expression may be considered a marker of invasion but is not significantly associated with hormone receptor status or prognosis [48,49].

### Colorectal cancer

Colorectal cancer has an incidence of 10% [24]. One of the earliest investigations of the role of the ABO blood group in colorectal disease investigated blood group differences in polyps of the colon. No association was detected between blood groups and adenomatous polyps, however the O blood group was found at a significantly higher frequency in patients with papillary adenomas, suggesting a different etiology for these two diseases [50]. In later studies evaluating the association between ABO blood group and colon cancer, no differences in blood group frequencies were detected between cases and controls [51,52]. Association of blood antigens with patient outcome is unclear with one study finding no association with survival differences in patients with metastatic colorectal cancer [53], while another study associated the AB blood type with improved survival [54]. Meta-analysis demonstrated a protective effect of the O blood type (OR: 0.89; 95% CI: 0.81–0.96), however, presence of A and/or B antigens was not associated with increased risk [23].

Evaluation of antigen expression in normal colon tissues revealed expression of antigens on the epithelial cells of the proximal colon, however, antigens were not expressed in cells of the normal distal colon. In colon carcinomas, 50% of tumors of the proximal colon demonstrated loss of antigen expression, although expression was retained in the adjacent tissues. Of note, in tumors of the distal colon, antigens, while undetectable in the adjacent tissues, were expressed in tumor cells [55,56]. Blood group antigens, are, however, expressed throughout the fetal period suggesting that re-expression of these histoblood group antigens are oncodevelopmental [57]. Although the role that histoblood ABO antigens play in colorectal tumorigenesis is not well understood, expression of A and B antigens has been associated with increased cellular motility and in a rat model, expression of the A antigen was associated with resistance to apoptosis, favoring tumorigenesis and metastatic spread [58,59].

### Prostate, liver & cervical cancers

Prostate, liver and cervical cancers accounts for 8, 6 and 4% of the global cancer incidence, respectively [24]. An analysis of multiple reports of the link between blood type and benign hyperplasia found no association with any of the blood types in this premalignant condition [60]. Lack of association between blood type and prostate cancer risk or survival has also been reported [61,62]. At the tissue level, while one study suggests that loss of antigen expression increases with tumor progression [63], a separate study found that lack of antigen expression of histoblood group antigens in prostate tumor tissue is not a marker of invasion [64].

Few studies have been performed evaluating the role of the histoblood ABO group in hepatocellular carcinoma (HCC). In a cohort of 6275 Chinese patients with chronic hepatitis B infection, male patients with the A blood group were at increased risk for developing HCC compared with those with blood type O (adjusted OR: 1.39; 95% CI: 1.05–1.83); an increased risk in males was also detected with blood group B, however, in female patients, those with blood types B or AB had decreased risk of HCC [65]. A separate study from Taiwan found females with the A blood group were at increased risk for developing liver cancer (hazard ratio: 1.69; 95% CI: 1.02–2.79) [66]. Evaluation of antigen expression in liver carcinoma tissues revealed no expression in HCC or hepatoblastomas and thus antigen expression in liver tumors cannot be used as a prognostic biomarker [67].

In a Japanese cohort of women, the A blood group was overrepresented in patients with cervical cancer [68]; this relationship was also seen in patients from Delhi, India [69]. In contrast, no relationship was found between blood group types and cervical cancer risk in patients from Nigeria or southeast Siberia. [70,71]. Evaluation of antigen expression within the tumor cells suggests that antigen expression decreases as tumors progress and antigen loss may be a marker of poor prognosis [72,73].

The association between blood group A and increased risk of stomach cancer is the strongest link for any of the cancers; the finding that patients with A blood type are more susceptible to *H. pylori*, a known stomach cancer causing agent, provides a mechanistic explanation for how a histoblood group antigen could promote tumorigenesis. The link for other cancers is much less clear. Confounding factors may include an excess of type O in blood donors compared with population controls or publication bias, with an underrepresentation of negative results in the literature [74], as well as the publication of studies based on small sample sizes. In addition, cancer is a multifactorial disease that may be significantly affected by germline variation as well as environmental exposures. Finally, the frequency of the ABO blood group varies globally, thus, differences in allele frequency of the ABO blood group must be considered when evaluating the role of ABO in disease.

## *ABO* gene polymorphisms & cancer

Despite the multitude of studies attempting to correlate ABO phenotype with cancer risk, the link between expression of histoblood group antigens and tumorigenesis was unclear for most tumor types evaluated. In 2009, an agnostic approach to identify genes associated with risk of developing pancreatic cancer further supported the association between the ABO blood group and cancer. Genotyping of 558,542 SNPs in 1896 patients with pancreatic cancer and 1939 controls revealed a significant association between SNP rs505922, located within the first intron of *ABO* [9]. The protective T allele of rs505922 is in linkage disequilibrium with the base pair deletion responsible for the O allele, suggesting that the A and B blood antigens may be associated with higher risk of pancreatic cancer. As with many of the other cancers, results of previous investigations of the role of the ABO antigens in pancreatic cancer were mixed, with some studies finding an association with expression of the A antigen [75] and others finding no association [76]; these discrepancies may be attributed to small sample sizes and retrospective study design. To this end, blood types were evaluated within the 107,503 eligible patients from the Nurses’ Health Study and Health Professionals Follow-up Study [77]. Evaluation of the two groups separately and combined revealed that patients with non-O blood types were significantly more likely to develop pancreatic cancer (adjusted hazard ratios for incident pancreatic cancer (A = 1.32 [95% CI:  1.02–1.72], AB = 1.51 [95% CI: 1.02–2.23] and B = 1.72 [95% CI: 1.25–2.38]). This targeted study supported the GWAS results with patients with the T/T genotype at SNP rs 505922 (or O blood group) having a protective advantage.

A number of follow-up studies have since been published. Evaluation of SNP rs505922 in patients treated at Memorial Sloan–Kettering Cancer Center and the Mayo Clinic validated the association between the C allele and increased risk of pancreatic risk (per allele OR: 1.65; 95% CI: 1–20–2.26), although this SNP was not associated with survival [78]. Using SNPs to determine blood types, variants associated with increased glycosyltransferase activity were associated with increased risk in the Pancreatic Cancer Cohort Consortium [79]. These findings have been validated in multiple populations, including American, Han Chinese, German, Japanese, Korean and Taiwanese [80–85]. Meta-analyses using both SNP rs505922 and blood types also support an association between non-O blood types and increased risk of pancreatic cancer [23,86]. Evaluation of blood groups across a spectrum of cancers revealed that the non-O blood types are associated with increased risk of exocrine pancreatic cancer but not with endocrine pancreatic cancer or any other cancer type studied, including stomach [19].

Evaluation of rs505922 has been performed with other cancers. In a group of Europeans with cholangiocarcinoma, allelic frequencies did not differ from those in controls; however, allele frequencies within the cohort were not in Hardy–Weinberg equilibrium, especially in patients with intrahepatic but not extrahepatic tumors [87], allowing for the possibility that this SNP is associated with a specific subtype of cholangiocarcinoma, as was seen for pancreatic cancer. To determine whether the ABO blood group was associated with breast cancer subtypes, SNP rs505922 was genotyped in 629 Caucasian women with invasive breast cancer, representing a variety of clinical and pathological tumor types. This data demonstrated that neither allele nor genotype frequencies varied significantly for any of the clinicopathological characteristics evaluated including age at diagnosis, tumor stage, size or grade, hormone, HER2 or lymph node status, intrinsic subtype, tumor type or patient outcome, suggesting that blood type is not associated with increased risk or less favorable tumor characteristics or prognosis in breast cancer [88].

## Modification of ABO phenotypes

Although associations between blood group and cancer risk have been evaluated at the phenotype (antigen expression) and genotype (SNPs in *ABO*) levels, expression of blood group antigens can be modified by other genes. For example, fucosyltransferase 1 (*FUT1*) encodes for H precursor substrate to which the A and/or B antigens attach; individuals homozygous for the hh genotype do not express the H substrate, resulting the O blood phenotype, regardless of *ABO* genotype [89]. This epistatic interaction may confound studies based on genotypes, as a fraction of patients predicted to be non-O blood types may have the O phenotype, overestimating the number of patients with A, B or AB blood types. This ‘Bombay phenotype’ is, however, rare, with a frequency of 1/10,000 in India and 1/250,000 in Caucasians [90]. Two other genes, *FUT2* and *FUT3*, affect the antigen expression in exocrine and mucosal secretions, with autosomal dominantly inherited genotypes resulting in secretors or nonsecretors. Secretor status may also confound studies of the role of the ABO blood group in cancer patients with non-O blood types may fail to express antigens within tissues of interest. For example, ABO antigens were not detected in lung cancers concomitant with decreased expression of FUT2 in tumor compared with adjacent lung tissues [91].

Associations between the H-, Secretor- and Lewis-antigens and cancer have been detected. For example, increased expression of FUT1 has been associated with increased propensity to metastasize in melanoma and advanced stage in ovarian cancer, while downregulation of FUT1 in a HER2-positive breast cancer cell line decreased proliferation [92–94]. DNA variants in FUT2 has been associated with increased risk of oral cancer in individuals who smoke and/or chew betel quid while in pancreatic cancer, secretor status was not associated with risk [95,96]. In an evaluation of variants within FUT loci variants within FUT2 and FUT3 were associated with interstinal- and diffuse-type gastric cancer, respectively [97]. Despite these efforts, no meta-analyses have been performed evaluating these modifiers of the ABO blood group phenotypes and more research is needed to understand the role of the blood group antigens in cancer.

## Discussion

More than 60 years have passed since the first report of a link between histoblood ABO antigens and cancer and despite the multitude of publications investigating the role of antigen expression in cancer risk or outcome, the original finding that individuals with blood type A were at increased risk for stomach cancer is supported by a recent meta-analysis which included 15,843 cases and 1,421,740 controls [18]. *H. pylori*, a known carcinogen for stomach cancer, adheres to the A antigen, which may explain how individuals with A blood type are at increased risk. For pancreatic cancer, the link between non-O blood type and cancer risk has been established both through blood types and by evaluating SNP rs505922; infection by *H. pylori* may also play a role in pancreatic cancer. altering the inflammatory response of the microenvironment and promoting tumor progression [98]. Data for other cancers are less clear, with some meta-analyses finding associations between non-O blood types and cancer risk while others found no association [19,23]. Although prospective studies with larger sample sizes may determine whether there is a relationship between blood type and cancer, improved understanding of the function of the histoblood ABO group antigens would provide mechanisms by which blood type could affect tumor etiology. Finally, one must consider how these data may be used clinically: risk associated with blood type may be affected by other genetic variants and numerous environmental factors, thus blood type is unlikely to be useful as a biomarker of risk, however, methods to block antigen expression may be effective in prevention or treatment of patients exposed to cancer-causing agents such as *H. pylori*.

## Conclusion

Additional work is needed to clarify whether blood types are associated with increased cancer risk and to determine how antigen expression affects tumorigenesis.

## Future perspective

The ability to definitely determine whether expression of antigens of the ABO blood group is associated with tumor etiology has been hampered by small sample sizes, different blood group frequencies by population and control populations enriched for the O blood type. In addition, detection of significant associations may be confounded by cancer risk factors such as genetic background or lifetime exposures. With technological improvements, such as next-generation sequencing and electronic health records, patient-centric databases may now be built that include a range of data types including genome sequences, metabolome and microbiome profiles and comprehensive health histories. For example, the Precision Medicine Initiative Cohort Program, sponsored by the National Institutes of Health in USA aims to recruit one million volunteers, healthy and with a variety of disease states and create a comprehensive epidemiological and molecular data base for each volunteer [99]. Projects such as this will allow for the collection of blood type as well as extensive molecular and epidemiological data and may provide sufficient power to determine whether the histo-ABO blood group is associated with cancer risk.

Although these modern, ‘Big Data’, approaches to science may conclusively determine whether ABO blood type is associated with risk of a variety of cancers, these associations do not explain how antigen expression may alter tumor susceptibility. Thus, these large-scale studies must be complemented by functional studies to determine whether ABO antigens have a function and if so, how that function may contribute to tumorigenesis. With the Precision Medicine Initiative Cohort Program slated to begin enrolling patients in 2016, our understanding of the role of blood group antigens in tumor etiology may be understood within the next decade.

**Table 1.** **Summary of data from published meta-analyses evaluating the relationships between ABO blood group types and cancer risk.**

**Study (year)**	**Tumor site**	**Study design**	**Results**	**Ref.**
Iodice *et al*. (2010)	Pancreatic	5,403 cases/125,893 controls	Decreased risk in patients with O compared with non-O blood type (SRR^a^, 0.79; 95% CI: 0.70–0.90)	[19]
Miao *et al*. (2014)	Breast	9.665 cases/244,768 controls	Increased risk for blood type A compared with type O (OR: 1.115; 95% CI: 0.992–1.254)	[20]
			Increased risk for blood type B compared with type O (OR: 0.983; 95% CI: 0.915–1.056)	
			Increased risk for blood type AB compared with type O (OR: 1.042; 95% CI: 0.881–1.231)	
Poole *et al*. (2012)	Ovarian	5,233 cases/6,837 controls	Moderately increased risk with A blood type compared with type O (OR: 1.09; 95% CI: 1.01–1.18; p = 0.03)	[21]
Risch *et al*. (2013)	Pancreatic	10,415 cases/869,044 controls	Increased risk with A blood type compared with type O in both cytotoxin-associated gene A nonendemic and endemic populations (OR_pooled_: 1.40; 95% CI: 1.32–1.49)	[22]
			Increased risk with blood types B and AB compared with type O with nonendemic populations (OR: 1.38; 95% CI: 1.16–1.64; and OR: 1.52; 95% CI: 1.24–1.85, respectively)	
Wang *et al*. (2012)	Gastric	1,045 cases/53,026 controls	Increased risk for blood group A compared with non-A (OR: 1.11; 95% CI: 1.07–1.15)	[18]
			Decreased risk blood group O compared with non-O (OR: 0.91; 95% CI: 0.89–0.94)	
Zhang *et al*. (2014)	Overall	82 case–control/seven cohort studies	Increase risk with A blood group compared with non-A (OR: 1.12; 95% CI: 1.09–1.16)	[23]
			Decreased risk O blood group compared with non-O (OR: 0.84; 95% CI: 0.80–0.88)	
Zhang *et al*. (2014)	Gastric	82 case–control/seven cohort studies	Increase risk with A blood group compared with non-A (OR: 1.18; 95% CI: 1.13–1.24)	[23]
			Decreased risk with O blood group compared with non-O (OR: 0.84; 95% CI: 0.80–0.88)	
Zhang *et al*. (2014)	Pancreatic	82 case–control/seven cohort studies	Increased risk with A blood group compared with non-A (OR: 1.23; 95% CI: 1.15–1.32)	[23]
			Decreased risk with O blood group compared with non-O (OR: 0.75; 95% CI: 0.70–0.80)	
Zhang *et al*. (2014)	Breast	82 case–control/seven cohort studies	Increased risk with A blood group compared with non-A (OR: 1.12; 95% CI: 1.01–1.24)	[23]
			Decreased risk with O blood group compared with non-O (OR: 0.90; 95% CI: 0.85–0.95)	
Zhang *et al*. (2014)	Colorectal	82 case–control/seven cohort studies	Decreased risk with O blood group compared with non-O (OR: 0.89; 95% CI: 0.81–0.96)	[23]
Zhang *et al*. (2014)	Ovarian	82 case–control/seven cohort studies	Increased risk with A blood group compared with non-A (OR: 1.16; 95% CI: 1.04–1.27)	[23]
			Decreased risk with O blood group compared with non-O (OR: 0.76; 95% CI: 0.53–1.00)	
Zhang *et al*. (2014)	Esophageal	82 case–control/seven cohort studies	Increased risk with B blood group compared with non-B (OR: 1.18; 95% CI: 1.04–1.32)	[23]
			Decreased risk with O blood group compared with non-O (OR: 0.94; 95% CI: 0.89–1.00)	
Zhang *et al*. (2014)	Lung	82 case–control/seven cohort studies	Decreased risk with B blood group compared with non-B (OR: 0.81; 95% CI: 0.66–0.95)	[23]
Zhang *et al*. (2014)	Nasopharyngeal	82 case–control/seven cohort studies	Increased risk for A blood group compared with non-A (OR: 1.17; 95% CI: 1.00–1.33)	[23]
			Decreased risk for O blood group compared with non-O (OR: 0.81; 95% CI: 0.70–0.91)	

OR: Odds ratio; SRR^a^: Summary relative risk.

**Executive summary**

The first reported association between ABO blood groups and cancer was reported in 1953; since that time, numerous studies with frequently conflicting results have been published.
Meta-analyses have detected a decreased risk of gastric, pancreatic, breast, ovarian, colorectal, esophageal and nasopharyngeal cancers for patients with O blood type.
Non-O blood types have been associated with significantly increased risk of gastric and pancreatic cancer.
The finding that non-O blood types are associated with increased risk of exocrine but not endocrine pancreatic cancer suggests that conflicting reports may be attributable to heterogeneity within tumor types.
Genome-wide association study studies provide agnostic support for a relationship between the ABO glycosyltransferase gene and cancer risk.
